# Daily Life Restrictions are Common and Associated with Health Concerns and Dietary Challenges in Adult Celiac Disease Patients Diagnosed in Childhood

**DOI:** 10.3390/nu11081718

**Published:** 2019-07-25

**Authors:** Heini Leinonen, Laura Kivelä, Marja-Leena Lähdeaho, Heini Huhtala, Katri Kaukinen, Kalle Kurppa

**Affiliations:** 1 Faculty of Medicine and Health Technology, Tampere University, 33014 Tampere, Finland; 2 Center for Child Health Research, Tampere University and Department of Pediatrics, Tampere University Hospital, 33014 Tampere, Finland; 3 Pediatric Research Center, Children’s Hospital, University of Helsinki and Helsinki University Hospital, 00029 Helsinki, Finland; 4 Faculty of Social Sciences, Tampere University, 33014 Tampere, Finland; 5 Department of Internal Medicine, Tampere University Hospital, 33014 Tampere, Finland; 6 Celiac Disease Research Center, Tampere University, 33014 Tampere, Finland; 7 The University Consortium of Seinäjoki, 60320 Seinäjoki, Finland

**Keywords:** celiac disease, daily life, experience, follow up, gluten-free diet, restrictions, transition

## Abstract

The prevalence and associated factors of daily life restrictions due to a gluten-free diet in adult celiac disease patients diagnosed in childhood are poorly known. We investigated these issues by collecting the medical data of 955 pediatric patients and sending questionnaires evaluating various health outcomes to the 559 patients who had reached adulthood. Of the 231 respondents, 46% reported everyday life restrictions caused by dietary treatment. Compared with those without restrictions, they more often had anemia at diagnosis (37% vs. 22%, *p* = 0.014), but the groups were comparable in other diagnostic features. In adulthood, patients with restrictions reported more overall symptoms (32% vs. 17%, *p* = 0.006), although the symptoms measured with the Gastrointestinal Symptom Rating Scale questionnaire were comparable. Despite strict dietary adherence in both groups, the experience of restrictions was associated with dietary challenges (34% vs. 9%, *p* < 0.001), health concerns (22% vs. 13%, *p* = 0.050), and lower vitality scores in the Psychological General Well-Being questionnaire. The groups did not differ in their current age, socioeconomic status, family history of celiac disease, general health or health-related lifestyle, the presence of co-morbidities, or regular follow up. Our results encourage healthcare professionals to discuss the possible health concerns and dietary challenges with patients to avoid unnecessary daily life restrictions, especially when young patients start to take responsibility for their treatment.

## 1. Introduction

During the past few decades, celiac disease has gradually become one of the most common chronic gastrointestinal diseases [1]. This is mostly due to improved case findings and at-risk group screenings, which have simultaneously resulted in earlier diagnoses with often a mild clinical presentation [2,3]. Although, in a broad sense, this is a positive trend, such a change may affect attitudes towards the diagnosis and treatment of celiac disease. A strict gluten-free diet is challenging and requires considerable effort in everyday life [4]. The availability and price of gluten-free products, as well as the possible difficulties caused by the diet in social, food-related situations, could impact negatively on the quality of life [4,5,6,7]. These challenges could eventually result in problems with dietary compliance—particularly if the symptoms at the time of diagnosis have been unspecific or negligible [6,7,8].

Age at diagnosis may have a major influence on how celiac disease and the gluten-free diet are experienced on an individual level [9]. Pediatric patients in particular constitute a special group, as the diet is usually initiated by the parents and maintained under their supervision until adolescence. Furthermore, patients diagnosed in childhood may not remember their initial symptoms or the reason for the celiac disease diagnosis [4,10], which could decrease the motivation to maintain the dietary treatment. Sufficient knowledge about celiac disease is therefore important to optimize coping with the disease and its long-term health outcomes. At present, however, there are limited data on how patients diagnosed as children experience the gluten-free diet in their daily life later in adulthood [11,12].

The aim of the present study was to evaluate the prevalence and associated factors of everyday life restrictions caused by long-term treated celiac disease. This was accomplished by comparing currently adult patients with and without reported restrictions caused by the gluten-free diet initiated in childhood.

## 2. Materials and Methods 

### 2.1. Patients and Study Design

**Figure 1:**
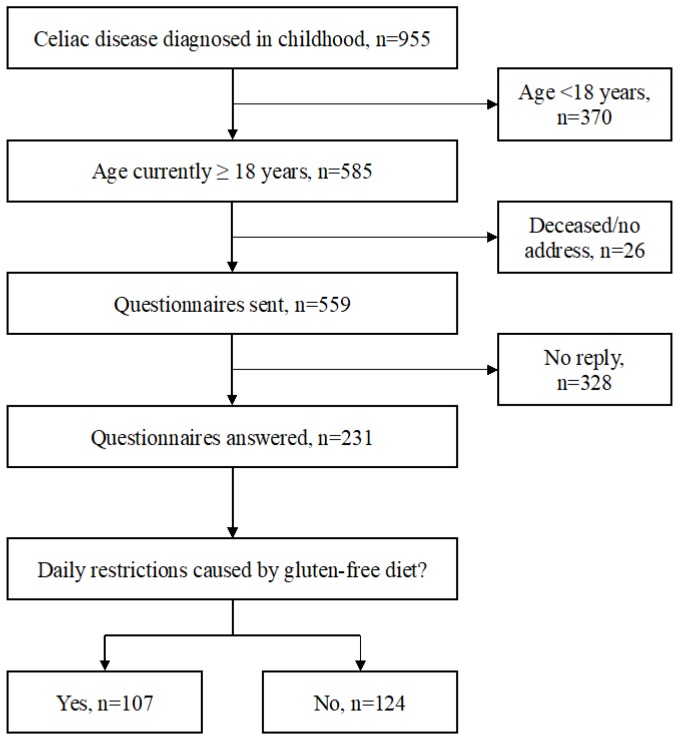
Flowchart of the data collection and study groups.

The study was carried out at Tampere University and Tampere University Hospital, Finland. The basis of the study cohort was a comprehensive research database of 955 biopsy-proven celiac disease patients diagnosed in childhood between 1966 and 2014 [12]. After excluding subjects who were at present below 18 years of age (*n* = 370), deceased (*n* = 7), or with missing contact information (*n* = 19), a specific study questionnaire and two validated questionnaires for gastrointestinal symptoms and quality of life were sent to the remaining 559 adult patients. The responders were divided into those who did and those did not report daily life restrictions caused by maintaining a gluten-free diet, and all study variables were compared between these two groups (Figure 1).

### 2.2. Diagnostic Features

The demographic data and the baseline clinical presentation, including symptoms and other possible signs of untreated celiac disease such as abnormal growth and the presence of anemia, were collected from the patient records. Anemia and growth disturbances were defined as findings, not symptoms. The main clinical presentation was further classified as: (1) gastrointestinal symptoms, e.g., abdominal pain, diarrhea, vomiting, constipation, and bloating; (2) extra-intestinal manifestation, e.g., dermatitis herpetiformis, growth disturbances, anemia, arthralgia, and neurologic symptoms; and (3) detection by screening at-risk groups, such as relatives of celiac disease patients and those with an associated autoimmune disorder. The severity of the symptoms was graded as: (1) none; (2) mild, occasionally disturbing symptoms; (3) moderate, more frequent and/or distracting symptoms; and (4) severe, continuous symptoms seriously disturbing daily life. Poor growth was defined as an abnormal deviation from the expected height and/or growth rate, as described elsewhere, [13] and anemia was defined as blood hemoglobin below the age- and sex-matched reference.

The severity of diagnostic histopathology was verified from the pathology reports. In our clinical routine, at least four duodenal biopsies are systemically taken upon esophagogastroduodenoscopy in each case of suspected celiac disease [14]. Mucosal damage is evaluated from representative and well-orientated mucosal specimens, and the lesion is further categorized as partial, subtotal, or total villous atrophy, which correspond approximately to Marsh-Oberhuber 3a–c [15]. 

### 2.3. Health and Treatment-Related Outcomes in Adulthood

The adult celiac disease patients were sent three separate questionnaires. The specific study survey was designed to evaluate the long-term health and treatment outcomes, and two well-validated surveys were used to measure the self-perceived gastrointestinal symptoms and health-related quality of life.

The specific study questionnaire consisted of questions regarding the current socioeconomic status, presence of children, family history of celiac disease, and a variety of life-style factors such as smoking and regularity of physical exercise. Furthermore, patients were asked about their current health experiences, including possible daily life restrictions due to a gluten-free diet, health-related concerns, presence of possible celiac disease-related symptoms, adherence to and challenges in maintaining a gluten-free diet, and the implementation of a follow-up for celiac disease. In addition, patients were asked about the possible presence of celiac disease-associated and other chronic disorders, regular medication, and complications such as fractures, miscarriages, and malignancies. Daily life restrictions due to a gluten-free diet were defined as a need to refuse an intended activity due to celiac disease. Patients were also asked about the situations where the restrictions were encountered. Health concerns refer to the possible experience of worries because of one’s state of health.

Self-experienced health was further classified as: (1) excellent or good; and (2) moderate or poor, and health concerns were classified as: (1) none; (2) minor; and (3) moderate or severe. The strictness of the dietary adherence was categorized as: (1) strict and (2) lapses more often than once a month. The implementation of the follow-up was classified as: (1) regular, denoting visits every 1–3 years; (2) occasional; and (3) no current follow-up. 

The Gastrointestinal Symptom Rating Scale (GSRS) was used to evaluate current gastrointestinal symptoms [16]. The questionnaire comprises 15 questions divided into five sub-dimensions, including diarrhea, indigestion, constipation, abdominal pain, and reflux. These are scored with a seven-point Likert scale from no symptoms (1) to the most severe symptoms (7). The values for each subcategory are calculated as the mean of the related items, and the total score is calculated as the mean of all 15 items.

The Psychological General Well-Being (PGWB) questionnaire was used to assess the self-perceived quality of life [17]. The validated survey contains 22 items covering six subdomains: anxiety, depressive mood, positive well-being, self-control, general health, and vitality. Each question is scored with a six-point Likert scale, with higher scores indicating a better quality of life. The total score ranges from 22 to 132 points, and the subdomain scores are calculated as the sums of selected questions.

### 2.4. Ethical Aspects

The study was conducted according to the Declaration of Helsinki. The Regional Ethics Committee of the Pirkanmaa Hospital District and the Department of Paediatrics, Tampere University Hospital, approved the data collection and questionnaire sending. Informed consent was obtained from all patients answering the questionnaires.

### 2.5. Statistical Analysis

The categorical data are presented as percentages and the quantitative data as medians with quartiles. Fisher’s exact test or the chi-squared test were used in statistical comparisons of the categorical variables, and the Mann–Whitney *U* test was used in comparisons of the numeric variables. A *p*-value < 0.05 was considered significant. All analyses were performed with SPSS version 24 (IBM Corporation, Armonk, NY, USA). The data were available for at least 90% of the patients in each variable unless otherwise stated.

## 3. Results

In total, 231 (41%) of the 559 adult patients responded to the questionnaires. The responders were more often women (69% vs. 52%, *p* < 0.001) and, based on medical records, they more often had a family history of celiac disease (56% vs. 44%, *p* = 0.029) and less coexisting type 1 diabetes (9% vs. 16%, *p* = 0.038). The responders and non-responders did not differ in age at diagnosis or year of diagnosis, main clinical presentation, severity of symptoms, degree of villous atrophy, or presence of poor growth and anemia.

A total of 107 (46%) of the 231 responders reported daily life restrictions caused by maintaining a gluten-free diet. These were most commonly encountered when eating at restaurants (72%), traveling abroad (38%), and visiting friends (30%). Subjects reporting restrictions more often had anemia than those without restrictions, but the groups were comparable in other disease features at childhood diagnosis (Table 1) and also in the median age at diagnosis (9.7 (quartiles 4.2, 13.0) years vs. 9.7 (6.0, 13.3) years, *p* = 0.603), and gender distribution (girls 67% vs. 72%, *p* = 0.460). The presence of anemia at childhood diagnosis also predicted a less common use of prescription medication in adulthood (30% vs. 46%, *p* = 0.028), but was not associated with other follow-up characteristics such as the employment status, family history, lifestyle, ongoing symptoms, health experiences, dietary adherence, challenges with the diet or presence of follow-up.

Upon current evaluation, patients with and without restrictions were comparable in median age (26.4 (21.7, 35.9) years vs. 27.7 (22.1, 30.2) years, *p* = 0.325) and time from the diagnosis (17.6 (12.0, 29.6) years vs. 18.6 (13.2, 31.6) years, *p* = 0.452), as well as socioeconomic status, membership of the celiac society, presence of children, family history of celiac disease, frequency of physical exercise, smoking, and use of medication (Table 2). In the detailed analysis, five patients reported a regular use of antidepressants, and none of the five reported restrictions caused by celiac disease. 

Subjects with restrictions had more health concerns and overall symptoms, such as abdominal complaints, arthralgia, skin symptoms and tiredness. One adult patient reported anemia and loss of hair, but she did not suffer from anemia at diagnosis. Those with restrictions found maintaining the gluten-free diet more challenging, whereas the groups did not differ in self-experienced general health, dietary adherence, or implementation of the follow up (Table 3). Health concerns, ongoing symptoms and challenges with the diet were not associated with gender or the presence of a regular follow-up. 

Based on the validated questionnaires, the patients with restrictions showed significantly worse PGWB vitality scores, whereas there were no differences in other aspects of the current self-perceived quality of life or gastrointestinal symptoms as measured by GSRS (Table 4). The prevalence of celiac disease-associated and other chronic comorbidities was similar for all diseases (45% vs. 47%, *p* = 0.771) and for each individual disease (Supplementary Table S1).

## 4. Discussion

We found that almost half of the celiac disease patients diagnosed as children reported everyday life restrictions due to a gluten-free diet in adulthood. The restrictive nature of celiac disease, particularly its dietary treatment, has also been recognized in a few earlier studies [18,19,20], but hitherto long-term data have been very scarce [21]. We consider it important to conduct studies focusing on adults diagnosed in childhood as a separate group, as the age at diagnosis may affect the experiences of celiac disease and the gluten-free diet substantially [9,22,23].

The daily life restrictions were associated with health concerns and a decreased experience of vitality. Accordingly, it has been observed that various restrictions in the social life and the psychological effects of dietary challenges may decrease the quality of life [20,24,25]. Although appropriately treated celiac disease patients in general have a good health status compared to patients of many other chronic diseases, the burden of a strict gluten-free diet can be considerable. For example, Shah and colleagues [26] reported that celiac disease patients find that their disease and its dietary treatment cause even more restrictions than dialysis for end-stage renal disease. It is also important to realize that, for currently somewhat unclear reasons, many celiac disease patients suffer from persistent symptoms even when following a strict gluten-free diet [27,28], and these symptoms may again affect their well-being negatively [29]. Correspondingly, we observed persistent symptoms more often among adult patients who experience restrictions. Symptoms can continuously remind the patient about the existence of celiac disease [27,29] and cause excessive monitoring of the diet due to the fear of an inadvertent gluten intake, further restricting daily life [30]. 

The patients reported that a gluten-free diet causes restrictions particularly when eating at restaurants, traveling, or visiting friends. Those reporting restrictions also found it harder to maintain the diet, despite their generally good adherence. Difficulties in food-related social situations when eating outside the home have been reported by celiac disease patients previously [18,19,22,23,31,32,33]. Challenges with the gluten-free diet may lead to the avoidance of situations that are considered difficult or, in the worst case, regular dietary lapses. Although it would seem logical that a less strict diet would at least make everyday life easier, difficulties in adherence have also been shown to be associated with a poorer quality of life [22,33]. Insufficient knowledge about the gluten-free diet could explain the avoidance of food-related situations and the feeling that the diet is unnecessarily difficult. Another explanation for these could be difficulties in the financial situation as gluten-free products are often more expensive than their gluten-containing counterparts [4]. However, the availability and selection of gluten-free products is in general good in Finland. These issues should be further discussed, and the patients should be educated both at diagnosis and later during follow-up visits.

On the other hand, only one-fourth of the celiac disease patients reported a regular long-term follow-up in adulthood, and this was not associated with the experience of restrictions. This is interesting, since one of the main reasons for recommending the routine follow-up of celiac disease is to support coping with the demanding diet [34,35]. The effectiveness of a systematic follow-up in facilitating the everyday monitoring of one’s own diet has been demonstrated previously in patients with type 1 diabetes [36]. Similarly, different social coping strategies and problem-solving skills, which could be taught during the follow-up, are thought to improve the everyday management of celiac disease [8,31]. The fact that here the presence or lack of a follow-up did not affect the experience of restrictions suggests the need for a more personalized approach, with a special emphasis on those with the highest risk for future problems.

Of the plausible factors predicting the later experience of restrictions, only the presence of anemia at diagnosis was found to have a significant association. Anemia has been associated with more severe celiac disease [37,38], but more studies are needed to decipher whether the observation has any clinical significance. There has been a concern that asymptomatic screening-detected patients in particular would consider the gluten-free diet burdensome, as it often has no apparent clinical benefits [4,7], but our findings do not support this hypothesis. However, we must remember that pediatric patients do not necessarily recall their clinical presentation in adulthood. Overall, it seems difficult to predict at diagnosis who will later experience restrictions and therefore needs particular surveillance. Adolescents in particular often consider themselves as outsiders and a “burden on others” because of their different diet [8]. The transition from pediatric to adult care might therefore be a good opportunity to identify those who could develop future challenges and benefit from intensified encouragement and peer support [39].

The major strength of the present study is the comprehensive long-term health data from adult celiac disease patients diagnosed in childhood. Furthermore, all relevant diagnostic data were verified from systemically maintained patient records, and the use of validated questionnaires for the quality of life and gastrointestinal symptoms increases the generalizability of the results [17,27,40,41,42]. The rather moderate response rate is a limitation, which may predispose to selection bias, likely toward results that are too positive [43]. However, this risk was reduced by the similarity of the responders and non-responders in the medical record data. Furthermore, we were able to also invite those patients to the study who do not make regular visits to health care. The fact that most of the current characteristics were self-reported has both advantages and disadvantages. For example, the patients’ own assessment may provide a more realistic picture of their quality of life compared to the physician’s evaluation [44], but, on the other hand, the reporting of comorbidities might be less reliable.

## 5. Conclusions

We found everyday life restrictions caused by a gluten-free diet to be common in adult celiac disease patients diagnosed in childhood. These restrictions were associated with challenges in maintaining the dietary treatment, persistent symptoms, and concerns about health. We therefore consider it important to discuss these issues during the follow-up to help dispel unnecessary worries, even when the dietary treatment seems to be successful.

## Figures and Tables

**Table 1:** Clinical characteristics of 231 children upon celiac disease diagnosis with and without later self-reported restrictions caused by a gluten-free diet (GFD) in adulthood.

	Restrictions due to GFD		
	Yes, *n* = 107%	No, *n* = 124%	*p*-Value
Anemia	37	22	0.014
Poor growth	35	27	0.258
Main clinical presentation			0.528
Screen-detected ^1^	20	19	
Extra-intestinal ^2^	31	25	
Gastrointestinal	50	57	
Severity of symptoms ^3^			0.149
None ^4^	32	27	
Mild	31	45	
Moderate	26	24	
Severe	11	4	
Severity of villous atrophy			0.653
Partial	34	32	
Subtotal	36	42	
Total	31	26	

^1^ E.g., celiac disease in the family or presence of an associated autoimmune disorder; ^2^ E.g., dermatitis herpetiformis, arthralgia, dental enamel defects, and neurologic symptoms; ^3^ Data available for 180 of the 231 patients; ^4^ Includes asymptomatic children with clinical signs such as poor growth or anemia. Due to rounding, the percentages may not add up precisely to 100%.

**Table 2:** Characteristics in 231 adults diagnosed with celiac disease in childhood with and without self-reported restrictions caused by a gluten-free diet (GFD).

	Restrictions due to GFD		
	Yes, *n* = 107%	No, *n* = 124%	*p*-Value
Employed ^1^	77	81	0.499
Member of celiac society	54	50	0.567
One or more children	45	40	0.436
Family history of celiac disease	56	65	0.180
Current or previous smoking	31	33	0.822
Prescription medication ^2^	38	42	0.782
Physical exercise			0.292
4−7 times per week	33	34	
1−3 times per week	53	46	
Less or no exercise	13	21	

^1^ Full-time or part-time; Data available for 181 of the 231 patients; ^2^ E.g., asthma medication, insulin, antidepressants, statins, and levothyroxine; contraceptives excluded. Due to rounding, the percentages may not add up precisely to 100%.

**Table 3:** Current experience of health and celiac disease in 231 adults diagnosed in childhood with and without self-reported restrictions caused by a gluten-free diet (GFD).

	Restrictions due to GFD		
	Yes, *n* = 107%	No, *n* = 124%	*p*-Value
Celiac disease-related symptoms ^1^	32	17	0.006
Maintaining the diet challenging	34	9	<0.001
Experience of current health			0.998
Excellent or good	82	82	
Moderate or poor	18	18	
Concerns about health			0.050
None	32	47	
Minor	46	40	
Moderate or severe	22	13	
Adherence to gluten-free diet			0.760
Strict diet	93	94	
Regular lapses ^2^	8	7	
Follow up of celiac disease			0.195
Regular	30	21	
Occasional ^3^	40	38	
None	31	40	

^1^ Based on self-assessment; ^2^ more than once a month; ^3^ less than every three years. Due to rounding, the percentages may not add up precisely to 100%.

**Table 4:** Current quality of life and gastrointestinal symptoms of 231 adults diagnosed with celiac disease in childhood with and without daily life restrictions caused by a gluten-free diet (GFD).

	Restrictions due to GFD		
	Yes, *n* = 107 Median (Q_1_, Q_3_)	No, *n* = 124 Median (Q_1_, Q_3_)	*p*-Value
Psychological General Well-Being ^1^			
Total score	103 (90, 112)	107 (96, 114)	0.188
Vitality	17 (15, 19)	18 (15, 20)	0.031
Anxiety	23 (19, 26)	24 (20, 26)	0.162
Depressive mood	17 (15, 18)	17 (15, 18)	0.206
Positive well-being	16 (14, 17)	16 (14, 17)	0.418
Self-control	16 (14, 17)	16 (14, 17)	0.134
General health	14 (11, 16)	14 (12, 17)	0.354
Gastrointestinal Symptom Rating Scale ^2^			
Total score	1.9 (1.5, 2.5)	1.9 (1.5, 2.3)	0.166
Diarrhea	1.3 (1.0, 2.0)	1.3 (1.0, 2.0)	0.084
Indigestion	2.5 (1.8, 3.3)	2.3 (1.8, 3.0)	0.237
Constipation	1.3 (1.0, 2.0)	1.3 (1.0, 2.0)	0.694
Abdominal pain	2.0 (1.3, 2.7)	1.7 (1.3, 2.3)	0.192
Reflux	1.0 (1.0, 2.0)	1.0 (1.0, 2.0)	0.167

Higher scores denote either ^1^ a better self-perceived quality of life or ^2^ more severe gastrointestinal symptoms. Q_1_, Q_3_: lower and upper quartiles.

## Supplementary Materials

The following are available online at https://www.mdpi.com/2072-6643/11/8/1718/s1, Supplementary Table S1: Presence of co-morbidities in 231 currently adult patients diagnosed with celiac disease in childhood with and without daily life restrictions caused by a gluten-free diet (GFD).
