# Supplementary material for: Daily Life Restrictions are Common and Associated with Health Concerns and Dietary Challenges in Adult Celiac Disease Patients Diagnosed in Childhood
Source: Nutrients. 2019 Jul 25;11(8):1718. doi: 10.3390/nu11081718 (PMC6723871; doi:10.3390/nu11081718)
Supplement: Supplementary file 1 [file nutrients-11-01718-s001.pdf]

nutrients

**Supplementary table.** Presence of co-morbidities in 231 currently adult patients diagnosed with celiac disease in childhood with and without daily life restrictions caused by a gluten-free diet (GFD).

| | Restrictions due to GFD | | |
|---|---|---|---|
| | **Yes, n=107** % | **No, n=124** % | ***p*-value** |
| Celiac disease-associated diseases | | | |
| *Thyroid disease* | 10 | 11 | 0.831 |
| *Type 1 diabetes* | 10 | 8 | 0.694 |
| Other conditions | | | |
| *Allergy*[1] | 44 | 40 | 0.591 |
| *Osteoporosis or fractures* | 27 | 24 | 0.613 |
| *Skin disease* | 18 | 14 | 0.458 |
| *Depression* | 14 | 12 | 0.651 |
| *Asthma* | 10 | 13 | 0.470 |
| *Miscarriages*[2] | 7 | 11 | 0.369 |
| *Other intestinal disease*[3] | 5 | 8 | 0.325 |
| *Hypertension* | 6 | 4 | 0.540 |
| *Rheumatic disease* | 6 | 3 | 0.307 |
| *Eating disorder* | 5 | 3 | 0.746 |
| *Cancer*[4] | 2 | 2 | 1.000 |

[1]E.g., allergies to animals, food, pollen, and medicine; [2]only in women; [3]E.g., Crohn's disease, ulcerative colitis, and gastric ulcer; [4]E.g., Hodgkin's lymphoma, non-Hodgkin's lymphoma, breast cancer, and brain cancer.

*Nutrients* **2019**, *11*, 1718; doi:10.3390/nu11081718 www.mdpi.com/journal/nutrients
